# Identification of Differentially Expressed MicroRNAs and Their Potential Target Genes in Adipose Tissue from Pigs with Highly Divergent Backfat Thickness

**DOI:** 10.3390/ani10040624

**Published:** 2020-04-05

**Authors:** Kai Xing, Xitong Zhao, Yibing Liu, Fengxia Zhang, Zhen Tan, Xiaolong Qi, Xiangguo Wang, Hemin Ni, Yong Guo, Xihui Sheng, Chuduan Wang

**Affiliations:** 1 Animal Science and Technology College, Beijing University of Agriculture, Beijing 102206, China; xingkbua@126.com (K.X.); buaqxl@126.com (X.Q.); xiangguo731@163.com (X.W.); nihemin@aliyun.com (H.N.); y63guo@126.com (Y.G.); 2 Key Laboratory of Animal Genetics, Breeding and Reproduction, Ministry of Agriculture, National Engineering laboratory for Animal Breeding, College of Animal Science and Technology, China Agricultural University, Beijing 100193, China; zhaoxitong0348@163.com (X.Z.); liuyb_0423@163.com (Y.L.); fengxiazhang@cau.edu.cn (F.Z.); 3 College of Animal Science and Technology, College of Tropical Agriculture and Forestry, Hainan University, Haikou 570228, China; tankoer@hainanu.edu.cn

**Keywords:** pig, miRNA, fat deposition, regulatory network

## Abstract

**Simple Summary:**

The role of microRNA in fat deposition is very important and not clearly understood. We detected 318 pig microRNAs (miRNAs), among high and low backfat tissue samples, by high throughput sequencing. Among them, 18 miRNAs were differentially expressed between the high and low backfat groups. Some of the differentially expressed miRNAs were involved mainly in lipid and carbohydrate metabolism, and glycan biosynthesis and metabolism. In addition, in silico analysis of the mRNA and miRNA transcriptomes, revealed possible regulatory relationships for fat deposition. In particular, three miRNA–mRNA pairs, miR-137–*PPARGC1A*, miR-141–*FASN*, and miR-122-5p–*PKM*, were identified as candidate key regulators of fat deposition. Our findings provide an important insight into miRNA expression patterns in backfat tissue of pig and new insights into the regulatory mechanisms of fat deposition in pig.

**Abstract:**

Fatty traits are very important in pig production. However, the role of microRNAs (miRNAs) in fat deposition is not clearly understood. In this study, we compared adipose miRNAs from three full-sibling pairs of female Landrace pigs, with high and low backfat thickness, to investigate the associated regulatory network. We obtained an average of 17.29 million raw reads from six libraries, 62.27% of which mapped to the pig reference genome. A total of 318 pig miRNAs were detected among the samples. Among them, 18 miRNAs were differentially expressed (*p*-value < 0.05, |log_2_fold change| ≥ 1) between the high and low backfat groups; 6 were up-regulated and 12 were down-regulated. Functional enrichment of the predicted target genes of the differentially expressed miRNAs, indicated that these miRNAs were involved mainly in lipid and carbohydrate metabolism, and glycan biosynthesis and metabolism. Comprehensive analysis of the mRNA and miRNA transcriptomes revealed possible regulatory relationships for fat deposition. Negatively correlated mRNA–miRNA pairs included miR-137–*PPARGC1A*, miR-141–*FASN*, and miR-122-5p–*PKM*, indicating these interactions may be key regulators of fat deposition. Our findings provide important insights into miRNA expression patterns in the backfat tissue of pig and new insights into the regulatory mechanisms of fat deposition in pig.

## 1. Introduction

Pig (*Sus scrofa*) is a vital agricultural animal for meat production [1]. Fat deposition is an important economic trait because it is correlated with carcass quality, meat quality, and consumer palatability [2]. Backfat thickness is a good indicator for fat deposition, and is usually measured within a certain period and at a specific age, then adjusted to a specified weight (100 kg). The backfat trait is highly heritable [3]. Selection for reduced backfat thickness has been effective [4] and is used directly in pig breeding [5].

MicroRNAs (miRNAs) are 18–22 base pair (bp) non-coding RNAs that are thought to regulate more than 60% of genes in almost all physiological and pathological processes [6]. Fat deposition is a complex biological process regulated by multiple factors, including miRNAs. In adipose tissue, miRNAs have been found to play important roles in adipocyte differentiation [7] and lipid metabolism [8]. For instance, miR-127 was found to be a negative regulator of adipogenesis by targeting the genes encoding mitogen-activated protein kinase 4 (MKK4) and homeobox C6 (HOXC6) in porcine adipocytes [9], and miR-302a inhibited adipogenesis by interacting with the 3′ UTR of peroxisome proliferator activated receptor gamma (*PPARγ*) mRNA [10].

Only 520 mature pig miRNAs (460 of which are not located in scaffolds) are recorded in the miRBase database (Release 22.1) (http://www.mirbase.org), which is much lower than the number of mature human miRNAs (2656) [11]. High-throughput sequencing can provide precise data on miRNA expression levels. A few studies of miRNAs in porcine adipose tissue have been reported, including differences in miRNA expression between sexes [12], among breeds [13,14], at different developmental periods [15], and in relation to backfat thickness [16,17]. However, the functions and molecular regulatory mechanisms of miRNAs in pig fat deposition are not clearly understood.

In this study, we used high-throughput RNA sequencing (RNA-seq) to reveal miRNA expression patterns in porcine backfat tissue, and to identify differentially expressed miRNAs between pigs with highly divergent backfat thickness. Furthermore, mRNA–miRNA interactions were analysed in silico, to define a potential regulatory network affecting pig fat deposition.

## 2. Materials and Methods

### 2.1. Animals

**Figure 1:**
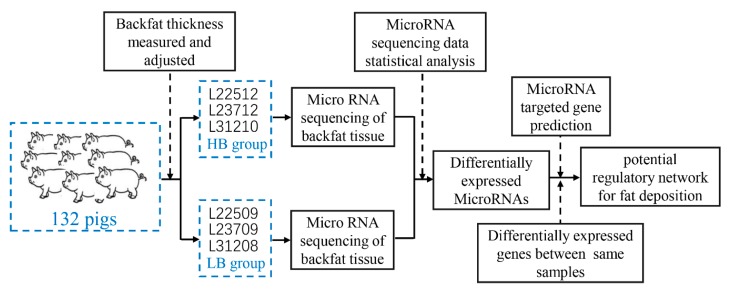
Experimental design used in this study. A total of 132 female Landrace pigs were separated into two groups. The HB group comprised pigs with high backfat thickness and included individuals L22512, L23712, and L31210. The LB group comprised pigs with low backfat thickness and included individuals L22509, L23709, and L31208. These six individuals were in the three HB/LB pairs of full-sibling pigs that showed extremes of backfat thickness and were selected for microRNA sequencing.

Using the same animals (female Landrace pigs) and methodology that we used in a previous study [18]; high backfat (HB) and low backfat (LB) pairs were selected as follows. The HB individual in a pair, had at least twice the backfat thickness as the LB individual, and the HB/LB pairs were full-siblings from the same litter. All 132 female Landrace pigs (185.53 ± 8.82 days old; 93.27 ± 18.64 kg) were kept in uniform and standard conditions and had ad libitum access to the same diet (Ninghe, China). Backfat thickness was measured between the 3rd- and 4th-last ribs using real-time B-mode ultrasonography (Honda Electronics, Toyohashi, Japan). Age, weight, backfat thickness, and pedigree information for the chosen pigs are shown in Supplementary Materials Table S1. Three of the HB/LB pairs that showed extremes of backfat thickness were selected for miRNA-seq (Figure 1). The backfat thickness was adjusted to 100 kg as follows:

(1) $$\mathrm{BFAD}=\mathrm{BF}\times \frac{13.983}{13.983+\left(0.126014\times \left(\mathrm{BW}-100\right)\right)}$$

where BFAD is backfat thickness adjusted to 100 kg, BF is backfat thickness, and BW is body weight.

BFAD was compared between the HB and LB groups using a t-test. BF and BFAD both showed significant differences between the two groups. The six selected pigs were slaughtered in a commercial abattoir (Beijing Huadu Sunshine Food Co., Ltd., Beijing, China), and their backfat tissue was collected. All efforts were made to minimize animal suffering during this study, which was approved by the Animal Welfare Committee of the China Agricultural University (permit number: DK996). Backfat adipose tissue between the 3rd- and 4th-last ribs was isolated and immediately frozen in liquid nitrogen for total RNA extraction.

### 2.2. RNA Extraction, Library Preparation, and Sequencing

Total RNA was extracted from the backfat tissue using TRIzol reagent (Invitrogen, Carlsbad, CA, USA) according to the manufacturer’s recommendations. The concentration and purity of the extracted RNA were evaluated using a Nanodrop 2000 spectrophotometer (Thermo Fisher Scientific, Waltham, MA, USA). RNA integrity was assessed using 1.5% agarose gel electrophoresis and the Agilent RNA Nano 6000 Assay Kit for Agilent 2100 Bioanalyzer (Agilent Technologies, Santa Clara, CA, USA) to ensure the samples were suitable for transcriptome sequencing. Six small RNA libraries were prepared from the total RNA using a TruSeq^®^ Small RNA Sample Prep Kit (Illumina^®^) following the manufacturer’s suggested protocol, then sequenced on an Illumina HiSeq 2000 platform (Illumina, San Diego, CA, USA) to obtain 50-bp single-end reads.

### 2.3. Statistical Analysis of the RNA-seq Data

Clean reads were obtained by removing adapters, low-quality reads, and reads <18 bp or >30 bp using the Fastx-toolkit (http://hannonlab.cshl.edu/fastx_toolkit). The obtained clean reads with high quality were used in the subsequent analyses. The clean reads were aligned to the Silva, GtRNAdb, Rfam, and Repbase databases, and the identified ribosomal RNA (rRNA), transfer RNA (tRNA), small nuclear RNA (snRNA), small nucleolar RNA (snoRNA), and other ncRNA sequences were removed. The remaining reads were mapped to the porcine reference genome (Sscrofa11.1, ftp://ftp.ensembl.org/pub/release-99/fasta/sus_scrofa/dna/) and searched against known miRNA sequences in miRBase 22.1 (408 miRNA precursors; 457 mature miRNAs) to identify miRNAs using Bowtie v 1.1.1 with the default parameters [19]. The expression levels of the miRNAs in the six libraries were normalized as transcripts per million (TPM) [20]. MiRNAs expressed at marginal levels (average of <1 TPM in the six samples) were removed because they are not useful and would add noise to the pairwise comparisons among the libraries. Differentially expressed miRNAs (DEMs) were identified using the edgeR package [21]. MiRNAs were considered to be differentially expressed when the false discovery rate (FDR) was ≤0.05 and the fold change (FC) was ≥2 or ≤0.5, calculated as |Log_2_FC| ≥ 1). DEMs also were detected between individual pigs in each pair of siblings using edgeR.

### 2.4. Prediction and Functional Analysis of DEM Target Genes in Silico

Because no porcine species is represented in either of the current miRDB (http://www.mirdb.org/) [22] or TargetScan (http://www.targetscan.org/) [23] databases, we used the homologous human miRNAs to predict putative targets. To reduce false positives, genes with a target scores of less than 80 in miRDB and a total context score of more than −0.40 in TargetScan were removed. Target genes that were predicted by both tools were retained for further analyses. Potential functions and pathways of the target genes were analysed using OmicShare tools (https://www.omicshare.com/tools/). The threshold for significant Gene Ontology (GO) terms and Kyoto Encyclopaedia of Genes and Genomes (KEGG) pathways was set as q-value < 0.05. In a previous study, we identified differentially expressed genes (DEGs) between HB and LB groups [18]. Target genes that were among the previously identified DEGs were considered as important candidate genes. Pearson and Spearman correlation coefficients between predicted miRNA–mRNA (target gene) pairs were calculated using R software. Significant negatively correlated DEM–DEG pairs were considered to have important regulatory relationships.

### 2.5. Quantitative Real-Time PCR

Quantitative real-time PCRs (qPCRs) were performed to confirm changes in miRNA expression levels between the HB and LB groups. Seven miRNAs were selected for validation. The same RNA samples that were used for the high-throughput RNA-seq were transcribed into cDNA using the stem-loop primer method for miRNAs. Porcine U6 snRNA was used as the internal control to correct for miRNA analytical variations [24]. The qPCRs were performed using the Power SYBR Green PCR Master Mix (Applied Biosystems, Foster City, CA, USA) in an ABI 7500 Real-Time PCR System (Applied Biosystems, Foster City, CA, USA). The qPCRs were performed by the Beijing SinoGene Scientific Co., Ltd.

## 3. Results

### 3.1. Overview of the miRNA Transcriptomes Profiles in the Six Libraries

An average of 17.29 million raw reads were obtained from the six libraries. After removing adaptors, contamination, and low-quality reads, 90.15% of the raw reads remained as clean reads. The clean reads were aligned against the porcine reference genome (Sscrofa11.1), and 62.27% of them were successfully mapped (Supplementary Materials Table S2). Among the clean reads, 4.99%, 0.13%, and 1.55% were identified as rRNAs, snoRNAs, and tRNAs, respectively.

### 3.2. Expression Patterns of miRNAs in Backfat Tissue

A total of 318 known porcine miRNAs (average TPM ≥ 1) were identified in the six libraries by searches against miRBase 22.1 (Supplementary Materials Table S3). The length distribution of the miRNAs showed that most of them were 21–23-nt in length, and the majority were 22-nt long. The number and expression levels of the miRNAs were similar in each library. Three known miRNAs (ssc-miR-1, ssc-miR-148a-3p, and ssc-miR-143-3p) had an average of approximately 1,000,000 reads each, and 20 known miRNAs had >100,000 reads each.

### 3.3. Identification of Differentially Expressed miRNAs

**Figure 2:**
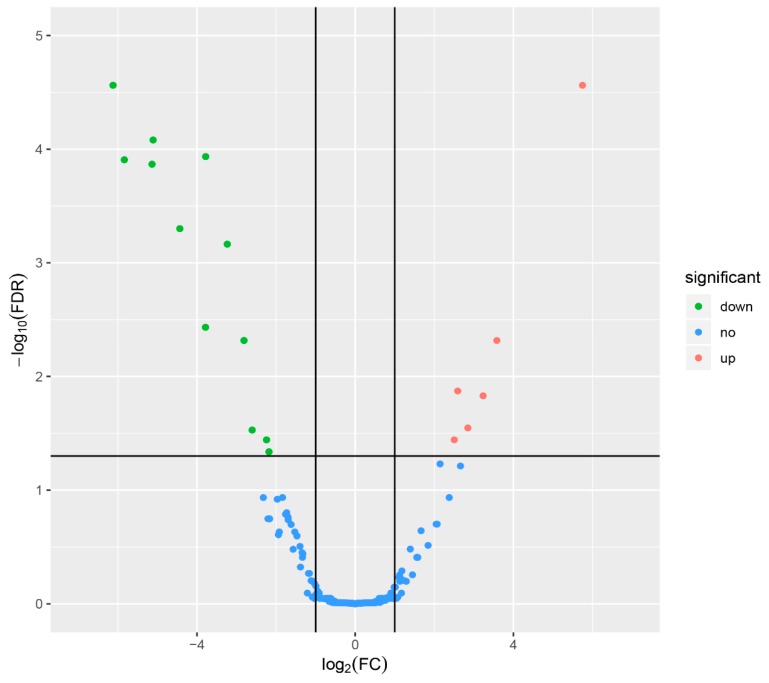
Scatter plot of the expression of the differentially expressed miRNAs between the high and low backfat groups. Red dots indicate up-regulated miRNAs in the high backfat group; green dots indicate down-regulated miRNAs in the low backfat group.

The expression levels of the miRNAs in the LB and HB groups were compared to identify DEMs using the edgeR package with a cut-off of FDR ≤ 0.05 and |log_2_FC| ≥ 1. A total of 18 DEMs were detected; 6 were up-regulated and 12 were down-regulated as detailed in Table 1 and Figure 2.

We identified 48, 12, and 0 DEMs using a threshold of q-value ≤0.05 and |log_2_FC| ≥1, and 128, 18, and 18 DEMs using a threshold of *p*-value ≤0.05 and |log_2_FC| ≥1 between individual pigs in each full-sibling pair. Six of the DEMs (ssc-miR-371-5p, ssc-miR-375, ssc-miR-202-5p, ssc-miR-183, ssc-miR-429, and ssc-miR-9) were common among the three pairs as detailed in Supplementary Materials Table S4. In this study, we focused our analysis on the DEMs obtained by treating the pairs as three biological replicates.

### 3.4. Target Gene Prediction and Functional Annotation

**Figure 3:**
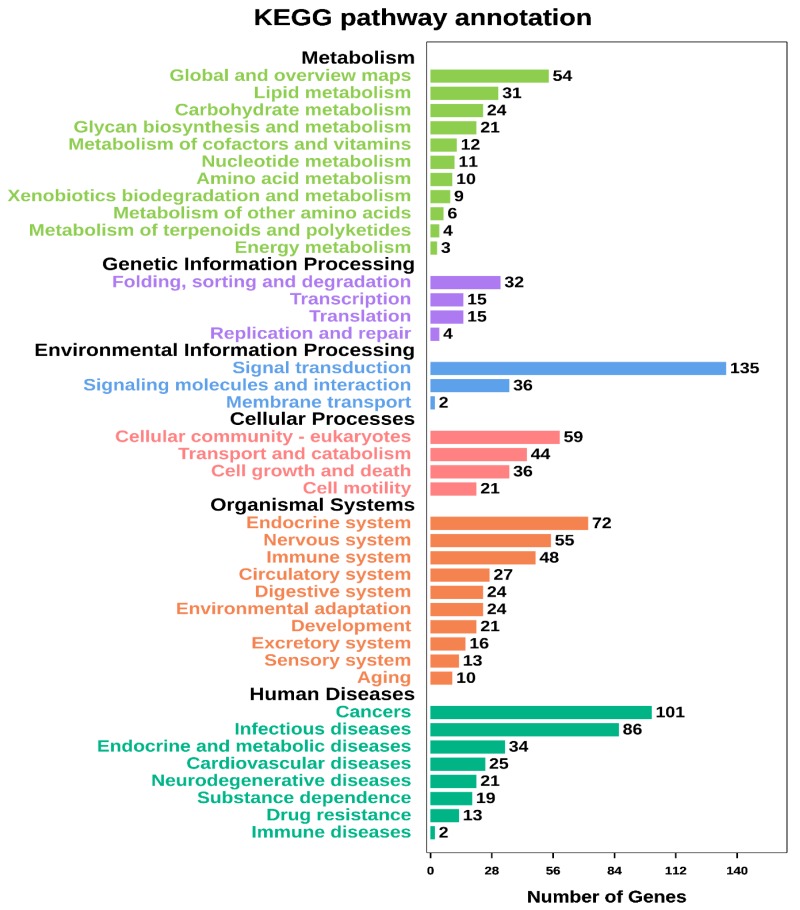
Enriched KEGG pathways of the predicted target genes of the differentially expressed miRNAs between the high and low backfat groups.

To elucidate the functions of the DEMs, we predicted their potential target genes. A total of 3600 and 2124 putative target genes were found for the 18 known DEMs using miRDB and TargetScan (Supplementary Materials Table S5), respectively. Among them, 942 genes were common in the two predictions, so these were selected for further analysis. The KEGG analysis annotated most of these target genes as involved in metabolism, including lipid and carbohydrate metabolism, and glycan biosynthesis and metabolism (Figure 3).

### 3.5. Proposed mRNA–miRNA Regulatory Relationship in Silico

In a previous study [18], we identified 564 DEGs in backfat tissue from pigs with HB and LB thickness phenotypes (Supplementary Materials Table S6), and 28 of them were among the 942 predicted target genes of the DEMs identified in the present study. An integrated analysis of the mRNA and miRNA expression profiles found 51 mRNA–miRNA pairs that were differentially expressed between the HB and LB groups. Twenty of these pairs showed opposite expression patterns between the two groups; however, only four of these pairs (miR-137–*PPARGC1A*, miR-141–*FASN*, miR-122-5p–*PKM*, and miR-122-5p–*CCNG1*) had significant negative relations in the Pearson correlation (correlation value ≤ −0.8; *p*-value ≤ 0.05). Two pairs (miR-141−*FASN* and miR-122-5p−*PKM*) had significant negative relationships in the Spearman correlation (correlation value ≤−0.8; *p*-value ≤0.05).

### 3.6. qPCR Validation

**Figure 4:**
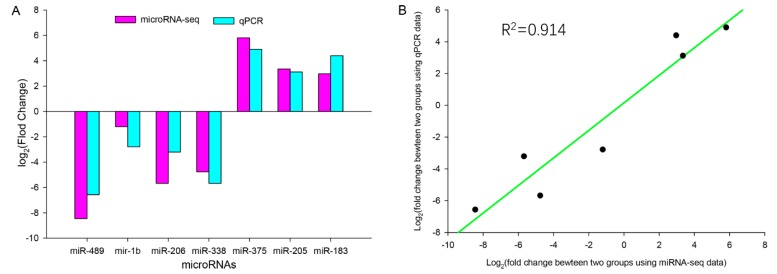
Validation of the genes miRNA expression patterns obtained from miRNA-seq data using qRT-PCR. (**A**) Assessment of Log2FC (fold changes (FC)) using the RNA-seq data and qPCR data for seven selected miRNAs. (**B**) The Relationship of Log2FC between both the high and low backfat groups using the RNA-seq data and qRT-PCR data (*p* < 0.05).

All the selected DEMs showed the same expression trends in the qPCR and miRNA-seq data (Figure 4A). The correlation of fold change between the qPCR and miRNA-seq expression levels was 0.914 (Figure 4B). These results indicated that the DEMs identified using the RNA-seq data were reliable.

## 4. Discussion

In this study, we characterised female Landrace pigs as having high or low backfat thickness. Landrace pig populations generally have low levels of fat deposition, including backfat thickness. However, we detected phenotypic variations in backfat deposition among the 132 experimental pigs (5.76 ± 1.75 mm) [18]. Significant differences in backfat thickness, but no differences in body weight, were observed between the pigs in the HB and LB groups, which implied that the differences in fat deposition were not caused by changes in body weight. We selected three HB/LB pairs of full-sibling pigs to assess differences in gene expression because we considered this strategy would reduce the noise associated with differences in the digenetic background and the possibility of false-positive results [16,25]. Using only three animal replicates for the HB and LB groups limits the conclusions that can be drawn from this study. However, the results provide valuable insights into the roles of miRNAs in regulating fat deposition that can be investigated in future studies. Some other studies that have reported miRNA–mRNA regulatory networks in animals have also used small numbers of animals [16,25,26].

High-throughput RNA-seq is a useful technology for analysing global miRNA expression patterns. We detected 403 known mature miRNAs among the six libraries using RNA-seq, which is a higher number than the previously reported numbers of miRNAs detected in porcine adipose tissue by RNA-seq (namely, 172 [27], 222 [28], 227 [15], 239 [14], 283 [12], and 309 [16]), but lower than the number (409) obtained by Davoli et al. [17]. Although miRBase does have some weaknesses, it has been used widely for identifying porcine miRNA and is a popular and reliable platform for miRNA researches [16,17,29,30]. Although MiRgeneDB (https://mirgenedb.org/) [31] and miRCarta (https://mircarta.cs.uni-saarland.de/) [32] provide more accurate and reliable miRNA annotations, miRNAs from porcine species are not included in the current MiRgeneDB. MiRCarta currently has miRNAs from 148 species, including 390 mature porcine miRNA sequences. All of these porcine miRNAs are also in miRBase. The top 10 most abundant miRNAs among all six samples were ssc-miR-148a-3p, ssc-miR-1, ssc-miR-143-3p, ssc-miR-99a-5p, ssc-let-7i-5p, ssc-miR-26a, ssc-miR-21-5p, ssc-miR-10b, ssc-let-7g, and ssc-let-7c, which implies that they play important roles in fat-related processes in adipose tissue, which is consistent with previous results. For example, miR-148a-3p was found to regulate adipocyte differentiation by targeting the gene that encodes lysine-specific demethylase 6b (KDM6B) [33], and miR-143-3p was shown to regulate differentiated adipocytes and several genes involved in insulin signalling at transcriptional or post-transcriptional levels in human in vitro differentiated adipocytes [34]. Let-7i-5p was shown to repress brite adipocyte function in mouse and human [35], but whether it plays important regulatory roles in adipogenesis was not determined.

Comparative analysis of miRNAomes from animals with opposite phenotypes is a useful method to investigate the functions of miRNAs. MiRNA expression profiles in adipose tissues from fatter and leaner porcine have been reported previously [12,16,17,24]. However, only four of the DEMs (ssc-mir-9-1-3p, ssc-miR-133, ssc-miR-183, and ssc-miR-1b) detected in the present study have been reported previously. The low consistency among the studies may be explained by differences in the experimental pigs that were used; for example, breed, body weight, age, and backfat. Several DEMs have been reported to play regulatory roles in fatty acid synthesis, lipid metabolism, and adipogenic differentiation. The expression levels of miR-133 in the fatter animals were found to be significantly lower than its expression levels in the leaner animals in three studies [16,17,24], which is consistent with the results of the present study. MiR-133 is known to inhibit preadipocyte differentiation from brown adipose and subcutaneous white adipose precursors to mature adipocytes by negatively regulating the transcription coregulator gene *PRDM16* [36]. Overexpression of miR-137 inhibited both human adipose tissue stromal cell proliferation and adipogenic differentiation by negatively controlling protein and mRNA levels of the cell division control protein 42 homolog (CDC42) [37]. MiR-31 has been shown to play an important role in the adipogenic differentiation process [38], and could influence body fat distribution by regulating the angiotensinogen (*AGT*) gene [39]. MiR-183, which was up-regulated in the HB group, was found to promote 3T3-L1 adipogenesis by inhibiting the Wnt/β-catenin signalling pathway [40].

To delineate the mechanisms of fat deposition, we constructed a potential regulatory network by an integrated analysis of the miRNA and mRNA transcriptomes. Several elements were predicted to play important roles in fat deposition and lipid metabolism. The level of fatty acid synthesis was shown to be higher in the backfat of fatter pigs than in the backfat of leaner pigs [18]. FASN is a key lipogenic enzyme and a rate-limiting step in de novo fatty acid synthesis in pig [41]. *FASN*, a predicted target gene of miR-141, was up-regulated in the HB group, and miR-141 was down-regulated (correlation value = −0.82, *p*-value = 0.047), which indicated that miR-141 may inhibit fatty acid synthesis by regulating *FASN* expression. We also found that up-regulation of *PPARGC1A* was linked to the down-regulation of miR-137 (correlation value = −0.90, *p*-value = 0.032). PPARGC1A was shown to play causal roles in regulating gluconeogenesis and adipogenesis [42]. Three potential miR-137 binding sites in the 3′ UTR of *PPARGC1A* have been reported [43]. Consistent with our results, *PPARGC1A* expression was found to be negatively correlated with miR-137 expression (*p*-value < 0.01) [44]. These results suggest that *PPARGC1A* may be down-regulated by miR-137 in adipose tissue and may be a new candidate gene affecting backfat thickness in pig. PKM is a pyruvate kinase that is involved in pyruvate metabolism, glycolysis/gluconeogenesis, and upstream pathways in lipid synthesis [45]. We found that the expressed levels of miR-122-5p and *PKM* were significantly negatively correlated (correlation value = −0.91, *p*-value = 0.043). MiR-122 has been reported to suppress glucose uptake by down-regulating *PKM* in breast cancer [46]. These findings suggest that the miR-122-5p–*PKM* interaction may be a new way of influencing backfat thickness in pigs. The candidate mRNA–miRNA regulatory pairs identified in the present study need to be further investigated to verify their functions in pig fat deposition.

## 5. Conclusions

In this study, we identified 18 DEMs from adipose tissue samples from pigs with high and low backfat thickness phenotypes. A comprehensive in silico analysis of the mRNA and miRNA transcriptomes identified negatively correlated mRNA–miRNA pairs, including miR-137–*PPARGC1A*, miR-141–*FASN*, and miR-122-5p–*PKM*, which may have important roles in fat deposition. The results of this study will facilitate the understanding of the molecular mechanisms involved in regulating fat deposition in pig.

## Figures and Tables

**Table 1:** The 18 differentially expressed miRNAs detected in Landrace pigs with low and high backfat phenotypes.

miRNA	Log_2_(TPM-HB)	Log_2_(TPM-LB)	Log_2_(FC)	*p*-Value	FDR
ssc-miR-375	11.54	5.24	5.75	1.25E-07	2.74E-05
ssc-miR-206	9.27	13.37	−6.12	1.77E-07	2.74E-05
ssc-miR-133b	4.35	7.30	−5.11	8.06E-07	8.30E-05
ssc-miR-885-5p	1.69	3.77	−3.78	1.50E-06	1.16E-04
ssc-miR-1b	14.20	18.01	−5.84	2.01E-06	1.24E-04
ssc-miR-133a-3p	8.78	11.85	−5.14	2.63E-06	1.36E-04
ssc-miR-486	5.78	8.27	−4.43	1.13E-05	5.00E-04
ssc-miR-137	1.64	4.55	−4.23	1.77E-05	6.85E-04
ssc-miR-9	7.66	11.47	−3.78	1.31E-04	3.69E-03
ssc-miR-141	9.90	5.72	3.58	2.08E-04	4.82E-03
ssc-miR-34c	3.43	5.98	−2.81	2.19E-04	4.82E-03
ssc-miR-31	6.54	3.40	2.59	6.51E-04	1.34E-02
ssc-miR-205	11.03	7.09	3.23	7.66E-04	1.48E-02
ssc-miR-183	8.95	5.34	2.85	1.56E-03	2.84E-02
ssc-miR-122-5p	5.37	7.46	−2.60	1.73E-03	2.96E-02
ssc-miR-493-5p	3.04	3.61	−2.24	2.29E-03	3.61E-02
ssc-miR-210	8.08	4.91	2.51	2.34E-03	3.61E-02
ssc-miR-323	3.90	5.38	−2.18	3.28E-03	4.60E-02

TPM-HB: the average of the TPM value in the HB group, TPM-LB: the average of the TPM value in the LB group, FC: fold change, FDR: false discovery rate.

## Supplementary Materials

The following are available online at https://www.mdpi.com/2076-2615/10/4/624/s1, Table S1: Age, weight, backfat thickness and pedigree information; Table S2: Summary of RNA-seq data alignment; Table S3: miRNA count in each sample; Table S4: Differentially expressed miRNAs between individual pigs in each pair of siblings; Table S5: Potential target genes predicted by miRDB and Targetscan; Table S6: Diiferentially expressed genes between HB and LB groups.

## References

[1] Sans P., Combris P. (2015). World meat consumption patterns: An overview of the last fifty years (1961–2011). Meat Sci..

[2] Suzuki K., Inomata K., Katoh K., Kadowaki H., Shibata T. (2009). Genetic correlations among carcass cross-sectional fat area ratios, production traits, intramuscular fat, and serum leptin concentration in Duroc pigs. J. Anim. Sci..

[3] Suzuki K., Irie M., Kadowaki H., Shibata T., Kumagai M., Nishida A. (2005). Genetic parameter estimates of meat quality traits in Duroc pigs selected for average daily gain, longissimus muscle area, backfat thickness, and intramuscular fat content. J. Anim. Sci..

[4] Gray R.C., Tribble L.F., Day B.N., Lasley J.F. (1968). Results of Five Generations of Selection for Low Backfat Thickness in Swine. J. Anim. Sci..

[5] Suzuki K., Kadowaki H., Shibata T., Uchida H., Nishida A. (2005). Selection for daily gain, loin-eye area, backfat thickness and intramuscular fat based on desired gains over seven generations of Duroc pigs. Livest. Prod. Sci..

[6] Bartel B. (2018). Metazoan MicroRNAs. Cell.

[7] Chen L., Song J., Cui J., Hou J., Zheng X., Li C., Liu L. (2013). microRNAs regulate adipocyte differentiation. Cell Boil. Int..

[8] Aryal B., Singh A.K., Rotllan N., Price N.D., Fernández-Hernando C. (2017). MicroRNAs and lipid metabolism. Curr. Opin. Lipidol..

[9] Gao Y., Wang Y., Chen X., Peng Y., Chen F., He Y., Pang W., Yang G., Yu T. (2019). MiR-127 attenuates adipogenesis by targeting MAPK4 and HOXC6 in porcine adipocytes. J. Cell. Physiol..

[10] Jeong B.-C., Kang I.-H., Koh J.-T. (2014). MicroRNA-302a inhibits adipogenesis by suppressing peroxisome proliferator-activated receptor γ expression. FEBS Lett..

[11] Kozomara A., Birgaoanu M., Griffiths-Jones S. (2018). miRBase: From microRNA sequences to function. Nucleic Acids Res..

[12] Mentzel C., Anthon C., Jacobsen M.J., Karlskov-Mortensen P., Bruun C.S., Jorgensen C.B., Gorodkin J., Cirera S., Fredholm M. (2015). Gender and Obesity Specific MicroRNA Expression in Adipose Tissue from Lean and Obese Pigs. PLoS ONE.

[13] Li H.-Y., Xi Q.-Y., Xiong Y.-Y., Liu X.-L., Cheng X., Shu G., Wang S.-B., Wang L.-N., Gao P., Zhu X.-T. (2012). Identification and comparison of microRNAs from skeletal muscle and adipose tissues from two porcine breeds. Anim. Genet..

[14] Wang Q., Qi R., Wang J., Huang W., Wu Y., Huang X., Yang F., Huang J., Xiaofeng H. (2017). Differential expression profile of miRNAs in porcine muscle and adipose tissue during development. Gene.

[15] Li G., Li Y., Li X., Ning X., Li M., Yang G. (2011). MicroRNA identity and abundance in developing swine adipose tissue as determined by solexa sequencing. J. Cell. Biochem..

[16] Liu X., Gong J., Wang L., Hou X., Gao H., Yan H., Zhao F., Zhang L., Wang L. (2019). Genome-Wide Profiling of the Microrna Transcriptome Regulatory Network to Identify Putative Candidate Genes Associated with Backfat Deposition in Pigs. Animals.

[17] Davoli R., Gaffo E., Zappaterra M., Bortoluzzi S., Zambonelli P. (2018). Identification of differentially expressed small RNAs and prediction of target genes in Italian Large White pigs with divergent backfat deposition. Anim. Genet..

[18] Xing K., Zhu F., Zhai L., Chen S., Tan Z., Sun Y., Hou Z., Wang C. (2016). Identification of genes for controlling swine adipose deposition by integrating transcriptome, whole-genome resequencing, and quantitative trait loci data. Sci. Rep..

[19] Langmead B., Salzberg S.L. (2012). Fast gapped-read alignment with Bowtie 2. Nat. Methods.

[20] Li B., Ruotti V., Stewart R.M., Thomson J.A., Dewey C.N. (2009). RNA-Seq gene expression estimation with read mapping uncertainty. Bioinformatics.

[21] Robinson M.D., McCarthy D.J., Smyth G.K. (2009). edgeR: A Bioconductor package for differential expression analysis of digital gene expression data. Bioinformatics.

[22] Wong N., Wang X. (2014). miRDB: An online resource for microRNA target prediction and functional annotations. Nucleic Acids Res..

[23] Lewis B.P., Shih I.-H., Jones-Rhoades M.W., Bartel B., Burge C.B. (2003). Prediction of Mammalian MicroRNA Targets. Cell.

[24] Chen C., Deng B., Qiao M., Zheng R., Chai J., Ding Y., Peng J., Jiang S. (2012). Solexa Sequencing Identification of Conserved and Novel microRNAs in Backfat of Large White and Chinese Meishan Pigs. PLoS ONE.

[25] Xing K., Zhao X., Ao H., Chen S., Yang T., Tan Z., Wang Y., Zhang F., Liu Y., Ni H. (2019). Transcriptome analysis of miRNA and mRNA in the livers of pigs with highly diverged backfat thickness. Sci. Rep..

[26] Huang M., Chen L., Shen Y., Chen J., Guo X., Xu N. (2019). Integrated mRNA and miRNA profile expression in livers of Jinhua and Landrace pigs. Asian-Australas. J. Anim. Sci..

[27] Liang Y., Wang Y., Ma L., Zhong Z., Yang X., Tao X., Chen X., He Z., Yang Y., Zeng K. (2019). Comparison of microRNAs in adipose and muscle tissue from seven indigenous Chinese breeds and Yorkshire pigs. Anim. Genet..

[28] Gaffo E., Zambonelli P., Bisognin A., Bortoluzzi S., Davoli R. (2014). miRNome of Italian Large White pig subcutaneous fat tissue: New miRNAs, isomiRs and moRNAs. Anim. Genet..

[29] Fleming D.S., Miller L. (2019). Differentially Expressed MiRNAs and tRNA Genes Affect Host Homeostasis During Highly Pathogenic Porcine Reproductive and Respiratory Syndrome Virus Infections in Young Pigs. Front. Genet..

[30] Yang K., Wang J., Wang K., Luo Y., Tang Q., Liu X., Fang M. (2020). Integrated Analysis of miRNA-mRNA Network Reveals Different Regulatory Patterns in the Endometrium of Meishan and Duroc Sows during Mid-Late Gestation. Animals.

[31] Fromm B., Domanska D., Høye E., Ovchinnikov V., Kang W., Aparicio-Puerta E., Johansen M., Flatmark K., Mathelier A., Hovig E. (2020). MirGeneDB 2.0: The metazoan microRNA complement. Nucleic Acids Res..

[32] Backes C., Fehlmann T., Kern F., Kehl T., Lenhof H.-P., Meese E., Keller A. (2017). miRCarta: A central repository for collecting miRNA candidates. Nucleic Acids Res..

[33] Tian L., Zheng F., Li Z., Wang H., Yuan H., Zhang X., Ma Z., Li X., Gao X., Wang B. (2017). miR-148a-3p regulates adipocyte and osteoblast differentiation by targeting lysine-specific demethylase 6b. Gene.

[34] Dahlman I., Belarbi Y., Laurencikiene J., Pettersson A.M., Arner P., Kulyté A. (2017). Comprehensive functional screening of miRNAs involved in fat cell insulin sensitivity among women. Am. J. Physiol. Metab..

[35] Giroud M., Karbiener M., Pisani D.F., Ghandour R.A., Beranger G., Niemi T., Taittonen M., Nuutila P., Virtanen K.A., Langin D. (2016). Let-7i-5p represses brite adipocyte function in mice and humans. Sci. Rep..

[36] Trajkovski M., Ahmed K., Esau C.C., Stoffel M. (2012). MyomiR-133 regulates brown fat differentiation through Prdm16. Nat. Cell Biol..

[37] Shin K.K., Kim Y.S., Kim J.Y., Bae Y.C., Jung J.S. (2014). miR-137 Controls Proliferation and Differentiation of Human Adipose Tissue Stromal Cells. Cell. Physiol. Biochem..

[38] Tang Y.-F., Zhang Y., Li X.-Y., Li C., Tian W., Liu L. (2009). Expression of miR-31, miR-125b-5p, and miR-326 in the Adipogenic Differentiation Process of Adipose-Derived Stem Cells. OMICS: A J. Integr. Boil..

[39] Machal J., Novak J., Hezova R., Zlamal F., Vasku A., Slaby O., Bienertová-Vašků J. (2015). Polymorphism in miR-31 and miR-584 binding site in the angiotensinogen gene differentially influences body fat distribution in both sexes. Genes Nutr..

[40] Chen C., Xiang H., Peng Y.-L., Peng J., Jiang S.-W. (2014). Mature miR-183, negatively regulated by transcription factor GATA3, promotes 3T3-L1 adipogenesis through inhibition of the canonical Wnt/β-catenin signaling pathway by targeting LRP6. Cell. Signal..

[41] Grzes M., Sadkowski S., Rzewuska K., Szydlowski M., Switonski M. (2016). Pig fatness in relation to FASN and INSIG2 genes polymorphism and their transcript level. Mol. Boil. Rep..

[42] Puigserver P., Spiegelman B.M. (2003). Peroxisome Proliferator-Activated Receptor-γ Coactivator 1α (PGC-1α): Transcriptional Coactivator and Metabolic Regulator. Endocr. Rev..

[43] Prastowo S., Widyas N., Ratriyanto A., Sohel M.H. (2018). In-silico identification of microRNAs potentially targeting the PGC1α gene that regulates bovine mitochondrial biogenesis. AIP Conf. Proc..

[44] Wei Q., Zhao L., Jiang L., Bi J., Yu Z., Zhao L., Song X., Sun M., Chen Y.Z., Wei M. (2018). Prognostic relevance of miR-137 and its liver microenvironment regulatory target gene AFM in hepatocellular carcinoma. J. Cell. Physiol..

[45] Sieczkowska H., Zybert A., Krzęcio-Nieczyporuk E., Antosik K., Koćwin-Podsiadła M., Pierzchała M., Urbański P. (2010). The expression of genes PKM2 and CAST in the muscle tissue of pigs differentiated by glycolytic potential and drip loss, with reference to the genetic group. Meat Sci..

[46] Fong M.Y., Zhou W., Liu L., Alontaga A.Y., Chandra M., Ashby J., Chow A., O’Connor S.T.F., Li S., Chin A.R. (2015). Breast-cancer-secreted miR-122 reprograms glucose metabolism in premetastatic niche to promote metastasis. Nature.

